# Editorial: Getting diagnosis right in child psychiatry: lessons from the pediatric bipolar disorder era

**DOI:** 10.3389/fpsyt.2025.1675976

**Published:** 2025-09-25

**Authors:** Peter Parry, Anne Cecilia Duffy, Glen Raymond Elliott, Glen Spielmans

**Affiliations:** ^1^ Childrens Health Queensland Clinical Unit, The University of Queensland, Brisbane, QLD, Australia; ^2^ College of Medicine and Public Health, Flinders University, Adelaide, SA, Australia; ^3^ Department of Psychiatry, Queen’s University, Kingston, ON, Canada; ^4^ Department of Psychiatry University of Oxford, Oxford, United Kingdom; ^5^ Children’s Health Council, Palo Alto, CA, United States; ^6^ Department of Psychology, Metropolitan State University, Saint Paul, MN, United States

**Keywords:** pediatric bipolar disorder (PBD), mania and bipolar disorder, child and adolescent psychiatric disorders, psychiatric nosology, developmental psychology, polypharmacy, parent child attachment, psychiatric assessment

This Research Topic, *Getting Diagnosis Right in Child Psychiatry: Lessons from the Pediatric Bipolar Disorder Era*, relates to a perennial problem in child and adolescent psychiatry – how to best diagnose, and then treat evolving psychopathology with a focus on emotional dysregulation and disruptive behavior in children and adolescents.

Pediatric Bipolar Disorder (PBD) is a controversial diagnostic construct that arose in the mid 1990s from a few influential US academic child and adolescent psychiatric centers. Over a million children and young teens were diagnosed, mostly in the USA but with some spread to Mediterranean and Latin American centers (1). Bipolar Disorder is a major mental illness with high heritability with typical onset as a depressive episode in late adolescence or early adulthood. It is associated with significant morbidity and mortality early in the course. Therefore, early detection is a laudable goal. However, the vast majority of youth diagnosed with PBD present with a chronic history of complex psychopathology including ADHD, pervasive developmental disorder, mood dysregulation and explosive temper in early childhood. The Kraepelinian description of a recurrent and spontaneously remitting course with distinctive episodes of depression and mania were entirely absent from PBD (2).

The lead topic editor’s doctoral thesis (3) examined the PBD phenomenon, concluding that a confluence of factors led to the PBD epidemic: desire for early detection of bipolar disorder before first full manic episode, a paradigm shift towards a simplistic biomedical reductionist perspective, influence of pharmaceutical industry research and CME funding and marketing to the public and parents’ groups, undervaluing of psychoanalytic, psychodynamic, family systems and biopsychosocial model perspectives, neglect of childhood trauma, maltreatment and insecure attachment, limitations of US health care corralling patients towards a medicalized pharmacotherapy approach, diagnostic upcoding due to managed care, and the appeal of a singular problem addressable with a medication fix [3, pp. 447-449]. The PBD overdiagnosis epidemic was associated with increased psychotropic polypharmacy and iatrogenic morbidity and mortality [3, pp. 137-141].

PBD as an “emblematic diagnosis for decontextualization” [3, p. 423] probed whether diagnostic labels are helpful, harmful, or distracting. Diagnoses that posit a disorder solely intrinsic to the child/teen likely lean towards pharmacotherapy strategies, in spite of recognition of cognitive behavior therapy and dialectical behavior therapy for emotional/behavioral dysregulation in adolescence. Diagnosing high rates of children – as young as toddlers and preschoolers – with mania (4–6) during the PBD era exhibited such informational and biomedical reductionism.

Psychiatric diagnosis was never meant to be based on merely a checklist of symptoms. Diagnosis should consider developmental history, psychosocial and family context, family history of psychopathology (genetic predisposition); further, this broadly-informed case conceptualization should be compared to research on the developmental trajectory of prototypical psychiatric illnesses (7). Focusing on symptoms alone in child and adolescent psychiatry (neglecting a developmental approach) can lead to misinterpretation of evolving psychopathology. This approach maps on to inadequate, unnecessary and at times harmful treatment.

A well-considered comprehensive diagnostic formulation can therefore guide the choice of treatments and therapies (and those which should be avoided so as not to cause harm), that would include parent-child dyadic therapy, family therapy, cognitive-behavioral and play therapy modalities, and school interventions, in addition to or alternative to pharmacotherapy.

This Research Topic has five papers that explore these aspects further.

McDermott et al. performed a retrospective analysis of a consecutive 2,131 children and teens, presenting over the five years from 2014 to 2019 to a Child & Adolescent Mental Health Service in northern Queensland, Australia. Only six were diagnosed with mania or bipolar disorder and were approximately two years older than the mean age of 12.6 years of the full cohort. A further 39 were diagnosed with cyclothymia but only two of these were under age 12 years and none progressed to bipolar disorder during the study period. These Australian results echo international diagnosis age of onset data presented by Clacey et al. (8) and are consistent with most longitudinal studies of high-risk offspring as to age of onset of the first manic episode (9).

Two papers address the excessive psychotropic polypharmacy characteristic of the PBD era and responsible for significant iatrogenic morbidity and mortality. Cosgrove et al. noted that the PBD era extended across the lifespan, in diagnoses like “treatment resistant depression” with related polypharmacy and often poor outcomes. They stated:

The medicalization of distress, the sedimented belief in “magic bullets,” and the push to “scale up” mental health treatment have contributed to the meteoric rise in the prescription of psychiatric drugs and of polypharmacy.

Their answer was a call for a paradigm shift to a “gentle medicine approach”, similar to geriatrics and family medicine where increasingly a “less-is-better approach” is implemented to reduce polypharmacy and focus on contextual factors in patients’ lives.

Theall et al. described a fictionalized case example of “snowball polypharmacy” for a young adolescent patient with an ADHD diagnosis and internalizing and externalizing symptoms. They explored the practicalities of ‘less-is-better’ with the findings from an expert focus group (6 psychiatrists, 2 pediatricians) on psychotropic deprescribing practice for children and youth with complex needs. They present a practical stepwise deprescribing approach of medication review, timing for medication reductions, careful monitoring and targeting one medication at a time, and considering the contextual settings and non-pharmacological interventions. The importance of slow tapering, particularly of antidepressants and antipsychotics was noted, and this is an aspect elucidated in the new *Maudsley Deprescribing Guidelines* (10).

Boudjerida et al. used the Delphi method to arrive at consensus views on how to assess and treat Disruptive Mood Dysregulation Disorder (DMDD). DMDD is a controversial diagnosis in that it was created by the DSM child and adolescent mood disorders committee for DSM-5 in 2013 to provide an alternative diagnosis that would reduce excessive diagnosing of PBD. Of 103 experienced clinical academics who were invited, 23 psychiatrists and psychologists across eight nations participated. Debate persisted about the validity of the DMDD construct. Clinical interview was preferred in assessment over use of standardized questionnaires and collection of history from multiple sources, parents, siblings, peers, teachers was emphasized. Various features of temper outbursts should be described systematically. Screening for physical, mental and neurodevelopmental disorders as differential or comorbid diagnoses should be undertaken. Context was emphasized by the experts, including whether the child was subject to maltreatment and negative life events. The classical four-step child psychiatric assessment process: interview parents and child together, parents alone, child alone, meeting with parents and child to sum up the situation, was favored.

There was greater consensus for psychosocial interventions:

Psychoeducation, behavior-focused therapy (e.g., dialectical behavioral therapy, chain analysis, exposition, relaxation) and systemic therapy (parent management training, family therapy, parent–child interaction therapy) met consensus.

Whereas there was little consensus for pharmacotherapy management of DMDD.

Finally, a paper on the life’s work of Dr Ed Levin (1931-2022), child and adolescent psychiatrist in Berkeley, California. Over the course of a long career, Levin stressed the need to consider attachment and developmental trauma in assessing and treating not only children and youth but also the elderly, as he discovered in later work consulting to a geriatric residential center (Parry).

Levin’s adherence to medical ethical principles put him at odds with what he perceived as conflicts of interest influencing child psychiatry and epitomized in the PBD epidemic (11). He championed the traditional epidemiology of bipolar disorder as exemplified in the findings of McDermott et al., advocated for exploring psychosocial context as the experts in the Delphi Consensus findings of Boudjerida et al., practiced the less-is-better pharmacotherapy as recommended by Cosgrove et al., and pioneered slow tapered deprescribing in his remarkable results in a youth residential center (12), as is now advocated by the experts surveyed by Theall et al. and new guidelines (10). These are the main lessons from the PBD era.

The controversy around PBD represents an important opportunity for the field to learn from past mistakes and move in the direction of more precise and developmentally informed diagnostic approaches. Often diagnosis in young people requires prospective longitudinal observation and should be predicated on collateral history (including from school and family members) and a detailed family psychiatric and social history. The DSM was never intended to be a stand-alone diagnostic checklist. Even when predicated on a comprehensive assessment, current psychiatric diagnoses are so heterogeneous they alone do not map to a specific treatment response. Adult psychiatry is becoming increasingly aware of the need for more homogenous subtypes to map to treatment, prognosis and biomarkers. For example, bipolar disorder that responds to long-term lithium treatment (13).

These articles re-emphasize that, especially in young patients, if the field relies solely on static diagnostic categories and symptoms without including other relevant information – such as childhood adversities, clinical course and psychosocial and developmental context – then it lacks the information needed for an effective evaluative and treatment approach. Such a simplistic approach overlooks the array of influences that affect a developing brain and observable behaviors. Especially difficult to capture in any symptom descriptive model are the remarkable changes, deleterious and beneficial, that occur from birth to adulthood across multiple biopsychosocial domains. While acknowledging the key role medications sometimes can play, simply writing a prescription – let alone numerous ones for the same patient – is not a viable solution to addressing an evolving mental illness or the child and adolescent’s emotional and behavioral issues.

## References

[1] Parry P Allison S Bastiampillai T. The geography of a controversial diagnosis: A bibliographic analysis of published academic perspectives on 'paediatric bipolar disorder'. Clin Child Psychol Psychiatry. (2019) 24:529–45. doi: 10.1177/1359104519836700, PMID: 30905170

[2] Duffy A Carlson G Dubicka B Hillegers MHJ. Pre-pubertal bipolar disorder: origins and current status of the controversy. Int J Bipolar Disord. (2020) 8:18. doi: 10.1186/s40345-020-00185-2, PMID: 32307651 PMC7167382

[3] Parry P. ‘Paediatric bipolar disorder’: why did it occur, the iatrogenic consequences, and the implications for medical ethics and psychiatric nosology. Flinders University (2021). Available online at: https://theses.flinders.edu.au/view/e8c15152-a279-4e61-88ce-e96080a908da/1.

[4] Biederman J Mick E Hammerness P Harpold T Aleardi M Dougherty M. Open-label, 8-week trial of olanzapine and risperidone for the treatment of bipolar disorder in preschool-age children. Biol Psychiatry. (2005) 58:589–94. doi: 10.1016/j.biopsych.2005.03.019, PMID: 16239162

[5] Luby JL Belden AC. Clinical characteristics of bipolar vs. unipolar depression in preschool children: an empirical investigation. J Clin Psychiatry. (2008) 69:1960–69. doi: 10.4088/jcp.v69n1216, PMID: 19192470 PMC2692382

[6] Moreno C Laje G Blanco C Jiang H Schmidt AB Olfson M. National trends in the outpatient diagnosis and treatment of bipolar disorder in youth. Arch Gen Psychiatry. (2007) 64:1032–9. doi: 10.1001/archpsyc.64.9.1032, PMID: 17768268

[7] Duffy A Malhi GS Carlson GA. The challenge of psychiatric diagnosis: Looking beyond the symptoms to the company that they keep. Bipolar Disord. (2018) 20:410–3. doi: 10.1111/bdi.12686, PMID: 30009578

[8] Clacey J Goldacre M James A. Paediatric bipolar disorder: international comparisons of hospital discharge rates 2000-2010. BJPsych Open. (2015) 1:166–71. doi: 10.1192/bjpo.bp.115.001933, PMID: 27703743 PMC4995564

[9] Duffy A Vandeleur C Heffer N Preisig M. The clinical trajectory of emerging bipolar disorder among the high-risk offspring of bipolar parents: current understanding and future considerations. Int J Bipolar Disord. (2017) 5:37. doi: 10.1186/s40345-017-0106-4, PMID: 29164495 PMC5698240

[10] Horowitz M Taylor DM. The Maudsley deprescribing guidelines: antidepressants, benzodiazepines, gabapentinoids and Z-drugs. John Wiley & Sons (2024). doi: 10.1002/9781394291052

[11] Levin EC Parry PI. Conflict of interest as a possible factor in the rise of pediatric bipolar disorder. Adolesc Psychiatry. (2011) 1:61–6. doi: 10.2174/2210676611101010061

[12] Levin EC. The challenges of treating developmental trauma disorder in a residential agency for youth. J Am Acad Psychoanal Dyn Psychiatry. (2009) 37:519–38. doi: 10.1521/jaap.2009.37.3.519, PMID: 19764849

[13] Duffy A Grof P. Longitudinal studies of bipolar patients and their families: translating findings to advance individualized risk prediction, treatment and research. Int J Bipolar Disord. (2024) 12:12. doi: 10.1186/s40345-024-00333-y, PMID: 38609722 PMC11014837

